# A journey with no roadmap—The need for validated criteria of the MS prodrome

**DOI:** 10.1177/13524585221135883

**Published:** 2022-11-08

**Authors:** Sharon Roman

**Affiliations:** The University of British Columbia, Vancouver, British Columbia, Canada

**Keywords:** Multiple sclerosis, biomarkers, epidemiology

## Abstract

**Background:**

A growing body of compelling evidence has emerged to validate a set of signs and symptoms that indicates the onset of disease before more typical signs and symptoms present to fulfill a diagnosis of MS. On 24 June 2021, a group of international researchers, patient advocates, and Society representatives led by Professors Helen Tremlett (University of British Columbia) and Ruth Ann Marrie (University of Manitoba) convened virtually for a workshop.

**Objective:**

Identify key gaps in knowledge, opportunities, and research priorities regarding the prodromal stage of MS.

**Methods:**

The group developed a new framework for MS that includes the stage of early signs and symptoms of MS—and outlined a roadmap to guide future research, with the “goal of preventing the progression to onset of typical symptoms of MS in those who present during the prodromal stage of MS”.

**Results:**

If high-risk individuals in the early stages of MS can be identified with a high degree of certainty, there is an opportunity to intervene and minimize the risk of progressing to typical MS symptoms and a diagnosis of MS.

**Conclusion:**

Standardized criteria must be developed, validated, and point of intervention found to better recognize, better diagnose, and better treat MS.

The euphemism “a journey” is firmly wedded to the patient experience of disease and navigating health care. There is an accompanying feeling of having taken a wrong turn, being lost, and needing guidance—needing a roadmap; so, it was that I found myself hospitalized for my first collision with multiple sclerosis (MS) at the age of 28 with sudden onset of dysarthria, ataxia, fatigue, and pseudobulbar affect. My travels through the myriad halls of health care and disability were launched with no foresight that day. At the time of my diagnosis, no framework—or roadmap—of the MS prodrome was established to help identify patients in the early stages of MS and prevent such disease impact.

MS is a neurodegenerative and inflammatory disease with genetic and environmental factors contributing to disease development. While a prodrome has been recognized in some neurological and autoimmune diseases, the MS prodrome has only recently been recognized with the emergence of a growing body of compelling data. An international group of researchers held a virtual workshop on 24 June 2021, led by Professors Helen Tremlett (University of British Columbia) and Ruth Ann Marrie (University of Manitoba), with the goal of identifying opportunities, key gaps in knowledge, and establishing research priorities to prevent “the progression to onset of typical symptoms of MS in those who present during the prodromal stage of MS.”^1^ A prodrome is an early set of signs and symptoms, often nonspecific, that indicates the onset of a disease before more classic symptoms occur—symptoms shown to occur 5–10 years before MS symptom onset in people who are subsequently diagnosed with MS; an eternity of questions for many patients.

The researchers revised the timeline of MS to incorporate a prodrome, and a framework for the stages of MS was proposed utilizing progress made in other neurodegenerative and autoimmune diseases, such as Parkinson disease^2^ (PD) and type 1 diabetes. The ability to identify those in the prodromal phase of MS with a high degree of certainty using validated prodromal criteria can aid in identifying potential points of intervention, minimizing the risk of progression to classical MS symptoms and a subsequent diagnosis of MS—offering an off-ramp to disease progression.

Where clear markers for disease and high risk meet lies the opportunity for early prevention measures—interventional therapy, such as a disease modifying therapy (DMT) or a novel neuroprotective drug to delay the early signs and symptoms from progressing to the onset of MS and a diagnosis of MS. Understanding and clearly identifying the stages of MS can guide prevention efforts. Optimal timing and duration of interventional therapy also needs to be established. Early intervention with a DMT has, for example, recently been shown to delay time from diagnosis to disability pension, further underscoring the importance of early recognition of MS, diagnosis, and treatment.^7^ “The hazard of receiving disability pension increased with increasing delay of treatment initiation.”^7^ The ramifications of early intervention are far-reaching; preventing loss of income, economic dependence, and social connection.

## A map for researchers

Several milestones must be achieved for this revised framework to become reality as set out in the roadmap. The key features of the MS prodrome are mostly unknown, including clinical signs and symptoms and the probability of developing classical MS when these are coupled with a person’s characteristics such as age, sex, or family history of MS. Identifying risk markers and likelihood ratios identifying how likely those markers are predictors of MS is needed to ascertain the strength of their associations with prodromal MS. The group drew a roadmap for future research with four main goals which are outlined in Table 1.

**Table 1.** Research goals.

**Capture population-based, age and sex-specific estimates for MS worldwide to serve as pre-test probabilities.** Utilizing the same approach as prodromal PD, age and sex-specific prevalence estimates can be used as prior probabilities for MS. The probability that an individual will develop MS before formal testing or evaluation corresponds with the prevalence of MS in the general population. This prior probability coupled with likelihood ratios of existing markers can be used to yield the likelihood an individual has prodromal MS. **Further identify characteristics of the prodromal stage of MS and develop new markers for disease**. Focus on specific informative populations, such as those with radiologically isolated syndrome (RIS). Until recently, neither RIS nor a prodromal phase for MS was even thought to exist,^3^ rather an individual moved through an indistinguishable continuum of normal to abnormal with nonspecific symptoms.^4^ While it remains controversial, evidence of a prodromal presence is mounting. Approximately half of people with RIS will go on to experience their first clinical event^5^ within 10 years; this group warrants particular attention. The evidence that Epstein–Barr virus might be necessary to develop MS^6^ yields another potentially valuable marker (a point of interest in my own journey); perhaps its absence may help rule out that a person is in the prodromal stage of MS. Populations, other than those with European ancestry, need investigation to determine the strengths of associations of putative markers as they may differ. Studying different populations might also offer opportunity to identify other new markers of prodromal MS. **Quantify the association between disease markers and probability of classical MS.** Markers of early disease activity can only be included in the criteria if we can measure the association between the disease marker and classical MS with a likelihood ratio. Of particular concern is the high rate of misdiagnosis of MS, making identifying markers that are highly likely to indicate MS critical. **Develop and validate criteria for the prodromal stage of MS for research purposes**. Once strong risk and early disease markers have been identified and validated, formal prodromal criteria, suitable for research purposes, can be developed to estimate the probability of developing classical MS over a defined time. With validated markers for those at high risk of developing typical MS, we can identify, for example, who might be eligible to enroll in clinical trials of interventions to prevent progression to typical MS. These criteria need to be substantiated with strong markers and the timing and duration of any interventional therapy established. A meaningful point of intervention needs to be determined and patient preferences and behavior considered (If I had been told I may be in the prodromal phase of MS, my point of acceptance of an intervention with potential harms may be different from someone else’s in a similar position—accepting these different preferences engages patients in shared-decision making in health care).

MS: multiple sclerosis; PD: Parkinson disease.

## The need for clear signs

Emerging evidence strongly supports the existence of an early, pre-clinical or prodromal stage of MS. Studies, especially clinical trials, require much longer than the standard two to three years, and are critical to the development of potential treatments and standardized criteria. Long-term commitments to studies that are capable of developing criteria to identify those in the prodromal stage with a high degree of certainty are needed. Early engagement of industry partners, regulators, advocacy groups, people with MS, and organizations who develop clinical care guidelines in these efforts is also needed.^1^

The significance of developing this milestone—a framework for MS—and long-term commitments to it cannot be overstated; preventing impacts such as mine with MS has far-reaching consequences. Achieving this landmark will enable us to better recognize, better diagnose, and better treat MS.

My journey through the byways of health care and rapid progression to disability would be empty if I did not raise my voice to issue a clarion call to action—so no one reads a doctor’s letter with the words “. . . there are some real concerns with respect to her future outlook,” as it relates to MS again. How much less the impact of MS might be, how much more guidance could be offered, if there was an established framework to allow early recognition and prevention of progression to a diagnosis MS—how much smoother the journey and less costly the ride.
